# Intratumoral Immunotherapy in Breast Cancer

**DOI:** 10.3390/vaccines13040429

**Published:** 2025-04-19

**Authors:** Camille C. Baumrucker, Nicole Harris, Susan Hoover, Brian J. Czerniecki

**Affiliations:** 1 Clinical Science Division, H. Lee Moffitt Cancer Center and Research Institute, Tampa, FL 33612, USA; camille.baumrucker@moffitt.org; 2 Department of Breast Oncology, H. Lee Moffitt Cancer Center and Research Institute, Tampa, FL 33612, USA

**Keywords:** breast cancer, immunotherapy, dendritic cell vaccines, tumor microenvironment

## Abstract

Breast cancer remains the most frequently diagnosed cancer and the second highest cause of cancer death in females. Metastatic recurrence that is resistant to traditional therapies presents a major challenge, necessitating the development of an innovative treatment strategy. Immunotherapy has gained popularity in the treatment of cancer, particularly melanoma, lung cancer, and more recently breast cancer. Major developments in immunotherapy have been made with a better understanding of the tumor microenvironment and how the microenvironment can be manipulated to induce an anti-tumor immune response. Intratumorally delivered immunotherapy can be used to create a local immune response. This review provides a comprehensive overview of intratumoral immunotherapy for breast cancer and its resultant changes in the tumor microenvironment. The discussed immunotherapeutics include oncolytic viruses, nucleic acids, innate immune agonists, bacteria, chimeric antigen receptor T cells, and dendritic cells. The review also evaluates completed clinical trials using these therapies. Lastly, the review offers future perspectives in the development of breast cancer immunotherapy.

## 1. Introduction

Breast cancer (BC) remains the most frequently diagnosed cancer in females worldwide with over 2 million women diagnosed annually and 670,000 deaths in 2022 [1]. Over 90% of BC deaths occur with metastatic disease [2], which is more frequently seen in triple negative breast cancer (TNBC) and HER2+ tumors than in luminal subtypes [3]. Treatment modalities for BC traditionally include a combination of surgical intervention, radiation, and systemic therapies. However, locally progressive cancers and metastatic recurrences present a major challenge as these tumors will inevitably display resistance to traditional therapies [2,3,4].

Immunotherapy has gained traction in the last decade and has become standard of care in melanoma and lung cancer, among others. The goal of immunotherapy is to stimulate the host’s own immune system to recognize and eliminate cancer cells. Initial immunotherapy drugs used in BC include immune checkpoint inhibitors (ICIs), specifically PD-1 and PDL-1 inhibitors. However, responses to these drugs remains variable due to the heterogeneity of breast tumors and possible lack of preexisting T-lymphocytes in some patients [5,6,7]. Additionally, systemically delivered ICIs have resulted in immune-related adverse events (AEs), including rash, infusion reaction, and hypothyroidism [8].

More recent advances in immunotherapy have recognized that the key to improving patient response rates lies in a more thorough understanding of the tumor microenvironment (TME) [9]. The breast has a complex immune microenvironment consisting of CD4+ helper T cells, CD8+ cytotoxic T cells, B cells, and natural killer (NK) cells [10]. BC has been considered an immunologically cold tumor as the tumor itself is infiltrated with immunosuppressive cells such as tumor-associated macrophages (TAMs), cancer-associated fibroblasts (CAFs), and myeloid derived suppressor cells (MDSCs). Infiltration of TAMs and CAFs promotes tumor growth and progression. This immunosuppressive environment likely contributes to resistance to traditional chemoradiation [11]. Prior research has shown that increased infiltration of TAMs and CAFs predicts a poor prognosis in BC while the presence of tumor-infiltrating lymphocytes (TILs) is associated with improved disease-free survival [12,13].

Direct intratumoral injection of immunotherapy has demonstrated promise in transforming the BC TME from “cold” to immunologically “hot” [11,12,13]. Intratumoral immunotherapy has the potential to activate dendritic cells (DCs), prompting their migration to the tumor draining-lymph node (TDLN) and subsequently inducing T- and B-cell responses [3]. Doing so may not only activate an anti-tumor immune response but also improve intratumoral T-cell infiltration and prime the tumor for subsequent therapies such as ICIs. Furthermore, local injection offers an alternative to reduce toxicities of systemically delivered therapeutics.

A variety of immunotherapeutics have been tested including oncolytic viruses, nucleic acid therapy, innate immune agonists, bacteria, chimeric antigen receptor (CAR) T cells, and DCs with various degrees of immune response and changes in the TME (Table 1). This review provides a comprehensive overview of trialed intratumoral immunotherapy for BC, focusing primarily on their impact on the TME and their clinical response. The subsequent sections discuss the lymph node response to intratumoral therapy and future perspectives.

## 2. Oncolytic Viruses

Oncolytic viruses (OVs) are a specialized class of immunotherapy in which native or genetically engineered viruses selectively replicate within tumor cells and ultimately lead to tumor lysis and regression [14,15,16]. The mechanism of action of OVs varies depending on the specific virus’s characteristics and dose. However, each OV has a method to enter the cell, promote viral replication, and ultimately result in oncolysis. Cell death releases various danger signals including tumor associated antigens (TAAs) and pathogen-associated molecular patterns (PAMPs), thereby activating antigen-presenting cells (APCs) and NK cells [17]. APCs like DCs go on to prime T cells in the TDLN and promote further trafficking of CD8+ T cells to sites of tumor growth, thus altering the TME [14,18,19].

### 2.1. Clinical Data

Talimogene laherparepvec (TVEC), a genetically engineered HSV1, is FDA-approved for melanoma but has shown promising results in BC as well. TVEC is manipulated to express the sequence for human GM-CSF, which promotes the activation and migration of DCs [14,15]. In melanoma, TVEC has been shown to increase DC influx while decreasing immunosuppressive cell phenotypes [20].

The oncolytic activity of T-VEC has shown promising results in early-stage clinical trials. A phase 1B study (NCT04185311) of six patients receiving intratumoral TVEC, ipilimumab, and nivolumab showed an increase in CD8+ T-cell infiltration as well as cytotoxic lymphocytes, monocytes, and NK cells. Of the six patients, one had a pathologic complete response (pCR), and three had a partial response. An increase in immune cell infiltration was associated with response to therapy [21]. Another phase 1B trial focused on patients with metastatic, unresectable, or locoregional recurrent HER2− negative BC. Nineteen patients received intratumoral T-VEC along with gemcitabine/carboplatin, nab-paclitaxel, paclitaxel, or endocrine therapy. No patients had a complete response, although 61% had a partial response. Responders demonstrated a decrease in circulating lymphocytes and in TIM3 expression, indicating T-VEC had an effect on the circulating immune landscape.

In a phase II trial (NCT02779855), patients with nonmetastatic TNBC received standard Adriamycin, cyclophosphamide, and Taxol chemotherapy as well as intratumoral TVEC. Tumors demonstrated an increased infiltration of CD3+CD8+ effector T cells and CD3+CD45RO+ memory T cells in the TME with 76% of patients having an absolute increase in CD8+ TILs. Immune cell infiltration correlated with clinical response; 16 of 37 (45.9%) patients had a residual cancer burden (RCB) 0 pCR [15].

Additionally, TVEC shows promise in inducing immunogenic changes outside of the TME. Early studies using TVEC in melanoma demonstrated increases in CD4+ and CD8+ T-cells in non-injected lesions, suggesting the therapy had effects outside the treated area [22]

T-VEC has also been explored in patients with metastatic or recurrent BC. A phase 2 trial of 11 patients with inoperable recurrence treated with T-VEC monotherapy was terminated prematurely due to disease progression. Investigators emphasized that this population may benefit from concurrent systemic therapy [23]. Patients receiving intra-hepatic T-VEC for liver metastases demonstrated only a 10% response rate. Adverse events in this trial were more severe than anticipated [24].

### 2.2. Safety Profile and Limitations

OVs are considered a generally safe and feasible intratumoral immunotherapy with patients experiencing mild AEs that are typically expected with injection of T-VEC. These include pain, erythema, edema, and flu-like symptoms. Severe AEs were limited but included fever requiring hospitalization and thromboembolic events. Grade 3-4 AEs like neutropenia, anemia, and thrombocytopenia are more likely attributable to the chemotherapy regimens administered to the patients in these trials.

The success of OVs is subject to the strength of the host’s own immune system. Dosing is highly variable due to viral proliferation upon administration, and removal of the virus is dependent on the host’s antiviral immune response, which some viruses are manipulated to evade [14]. A delicate balance must be achieved between promoting an anti-tumor immune response and limiting a neutralizing antiviral response [17].

### 2.3. Summary

OVs show promise in the treatment of early-stage BC with considerable evidence of changes in the TME and the systemic immune response. The immunologic changes correlate with positive clinical responses. The most promising evidence has been shown in TNBC patients, although there are limited data in early-stage hormone-positive disease. The use of T-VEC for more advanced BC must be further investigated. Ongoing clinical trials are focused on exploring additional types of viruses and primarily include patients with metastatic TNBC.

## 3. Nucleic Acids

Nucleic acid-based therapies include DNA- and RNA-based therapies that are engineered to encode a therapeutic gene of interest that will direct expression of the intended protein. These therapies offer easy reproducibility and versatility. DNA therapy consists of plasmids, circular DNA molecules that encode the protein of interest. Plasmids must be delivered into the nucleus where their genes can be transcribed. To successfully introduce the plasmid into the nucleus, assistance from physical forces such as electroporation may be required. RNA therapy more specifically includes mRNA-based therapies that can be delivered directly into the cytoplasm, although they are more unstable upon administration [25,26]. Even after successful administration, these therapies ultimately rely on adequate signaling pathways and activation of endogenous DCs.

### 3.1. Clinical Data

#### 3.1.1. DNA-Based Therapy

Plasmid IL-12 was studied in a phase 1 trial of patients with metastatic TNBC. Patients received three intratumoral injection followed immediately by electroporation. Four patients demonstrated an increase in CD8+ TILs but saw no increase in NK cells; this was associated with increased expression of genes involved in chemotaxis, T-cell activation, antigen presentation, and cytokine signaling pathways. Increases in CXCL 9/10/11/CXCR3 signaling pathways and increased expression of PD-1/PD-L1 were observed. No data on objective responses were reported; however, the researchers did note that one patient who was previously unresponsive to anti-PD-1 therapy went on to receive additional anti-PD-1 therapy after the trial, with an improved clinical response [27].

A phase 2 clinical trial further evaluated plasmid IL-12 in combination with pembrolizumab in inoperable recurrent or metastatic TNBC. The objective response rate (ORR) was 27.3%, and investigators observed a regression of untreated lesions as well. No grade 3-4 AEs attributable to the plasmid were observed. Decreases in myeloid derived suppressor cells (MDSCs) were observed in peripheral blood [28].

#### 3.1.2. mRNA-Based Therapy

mRNA-2752 was evaluated in a phase 1 trial for the treatment of solid tumors including TNBC. This therapy encodes proinflammatory cytokines IL-23 and IL-36 gamma and OX40L T-cell stimulator. Objective responses in BC are not published yet. Researchers did find that intratumoral delivery of mRNA-2752 increased levels of IL-23 and IL-36 gamma protein expression as well as their downstream cytokines in the tumor and in peripheral blood. Elevations of IFN-gamma, TNF-alpha, PD-L1, and markers of T-cell infiltration were observed, and researchers concluded this may provide a rationale for combination therapy with PD-L1 inhibitors [29].

### 3.2. Safety Profile and Limitations

• The safety profile of nucleic acid-based therapy seems to be reasonable; however, there are relatively few studies. No severe AEs were attributable to plasmid IL-12 [27,28]. However, this study only evaluated a single dose of the therapy and included a limited number of patients. mRNA-2752 had a single dose-limiting toxicity (DLT) at 8 mg, characterized as cytokine-release syndrome [29].
• Studies of nucleic acid-based therapy have generally focused on advanced TNBC, and their utility is unknown in patients with early-stage cancers. Moreover, advancements in delivery systems for DNA-based therapies are required to enhance their immunogenicity and potency. Continued research in tumor antigen selection will improve efficacy and personalization of these therapies.

### 3.3. Summary

• Nucleic acids therapies are not yet well-studied in the BC population. Early studies do show some evidence of an immune response; however, this is not well clinically correlated with objective tumor regression. These therapies seem to have a generally acceptable safety profile and may work better in combination with pembrolizumab. Ultimately, more research is necessary to further explore their use in BC.

## 4. Innate Immune Agonists

Innate immune agonists have gained favor as a strategy to activate the body’s first line of defense against cancer. Two pathways of interests include toll-like receptors (TLRs) and the stimulator of interferon gamma (STING) pathway [30].

### 4.1. Clinical Data

#### 4.1.1. Toll-like Receptors

TLRs can activate NK cells and inhibit MDSCs; however, they have also been shown to have pro-tumor effects [31]. In a phase I trial, the TLR9 agonist IMO-2125 given intratumorally into solid tumors, including BC, induced changes in the TME. Increases in MHC I and II expression, IFN-gamma gene expression, and the IFN pathways were identified. Like other immunotherapies, mild AEs were experienced, most frequently fever and chills [32]. Prior studies using intratumoral TLR9 in lymphoma have also demonstrated a decrease in size of non-injected tumor sites, suggesting this therapy may too have an abscopal effect [33].

#### 4.1.2. STING Pathway

The STING pathway promotes IFN production and DC activation, which can thereby induce a CD8+ T-cell response. Intratumoral injection of the STING agonist E7766 has been studied in a clinical trial of solid tumors including BC. No dose-limiting toxicities were recorded. No clinical response data are available, and the study was prematurely terminated for reasons unrelated to safety or clinical response [34].

### 4.2. Safety Profile and Limitations

The TLR9 agonist IMO-2125 appears to have a reasonable safety profile with only mild, expected AEs. However, it is unclear how many participants in this study had BC. No data are available regarding STING agonist E7766, so safety cannot be assessed.

Similar to nucleic acid therapies, innate immune agonists rely on proper activation of signaling pathways and successful maturation of monocytes into cDC1s. Furthermore, these agonists have been shown to activate responses in both cancer cells and immune cells, creating challenges in limiting off-target effects [35].

### 4.3. Summary

Overall, there are limited data supporting the use of innate immune agonists in the treatment of BC. IMO-2125 provides some evidence that it induces changes in the TME, but it is unclear if these changes were seen in BC specifically as this trial broadly included solid tumors. Prior research supports TLR9’s abscopal effect, although this has not yet been demonstrated in BC. Further preclinical studies are necessary to narrow the effects of innate immune agonists to the tumor while minimizing off-target effects.

## 5. Bacteria

Bacteria offer another genetically modifiable, versatile immunotherapeutic. A variety of bacteria have been studied in this context; however, bacterial species interact with the TME in different ways and can result in changes that promote or suppress tumor growth [36].

### 5.1. Clinical Data

Clostridium *noyvi*-NT (non-toxic), an attenuated, spore-forming anaerobe, has been explored as a cancer treatment because of its ability to replicate exclusively in the hypoxic tumor environment and induce tumor lysis. Of 24 patients with advanced solid tumors (two with BC) receiving a single intratumoral injection, 42% had bacterial spore germination and resulting cell lysis. Three patients with germination had evidence of increased T-cell infiltration. Of the two patients with BC, one continued with stable disease; the second patient was deemed unevaluable. Toxicities were non-negligible, including grade 4 sepsis and gas gangrene [37].

An ongoing phase 1 trial is assessing the utility of intratumoral Clostridium *noyvi*-NT with pembrolizumab. Sixteen patients started the study with at least two with BC. There were no DLTs; 10 of 16 patients experienced disease progression. The ORR was 25%, although none were in the BC patients [38]

### 5.2. Safety Profile and Limitations

As demonstrated by the completed phase 1 trial, bacterial immunotherapy can result in more severe AEs than the previously discussed therapies. Bacterial immunotherapies present a unique toxicity due to their risk of off-target effects and symptomatic infections [39]. Patients receiving immunosuppressive chemotherapy may be at higher risk, thus limiting use in cancer patients.

Owing to its affinity for the hypoxic TME, most bacteria colonize in the central, most hypoxic area of the tumor. Less bacteria are present on the periphery of the tumor, resulting in suboptimal anti-tumor responses. Furthermore, this variability in growth presents a challenge in dosing bacterial therapy. Since the mechanism by which this therapy results in lysis is poorly understood, it is challenging to predict which patients and tumors will mount an anti-tumor response [40].

### 5.3. Summary

Bacterial immunotherapy presents several challenges that must be overcome before it can be used clinically. A better understanding of its mechanism will allow more precise dosing and help better control severe AEs. Despite some promising immunologic results, clinical response remains largely unknown especially in BC patients.

## 6. CAR T Cells

CAR T-cell therapy has displayed therapeutic success in hematologic malignancies, prompting exploration in solid tumors including BC. It relies on autologous T cells that are manipulated to attack a specific tumor antigen such as c-Met, HER2, or EGFR [41,42].

### 6.1. Clinical Data

A phase 1 clinical trial focused on intratumoral c-Met-CAR T cells in metastatic BC. Researchers deemed this to be a safe and feasible approach. After injection, tumors showed evidence of TME changes with infiltration of PMNs, primarily CD4+ cells and mononuclear immune cells, although there was no evidence of NK cell or DC infiltration. CAR mRNA was detectable in the peripheral blood at 20 min in two patients and at 2 h in one patient post-injection though not after day 1. On a larger scale, tumors developed necrosis and hemorrhage after injection. There were reported grade III AEs, though these were not attributed to the CAR T-cell therapy [41,43]

### 6.2. Safety Profile and Limitations

Thus far, CAR T-cell therapy has not shown the same promise in BC as it did in hematologic malignancy. Although the reason for the lack of efficacy is not fully understood, it is hypothesized that the immunosuppressive TME is not suitable for sustained T-cell survival [44]. It has been hypothesized that combination therapy with PD-1 blockade may improve T-cell persistence [45]. A second major challenge in the generalizability of CAR T-cell therapy is managing off-target effects [46]. Several clinical trials looking at this therapy were stopped early when it was found that both malignant and healthy tissue expressed the target antigen, resulting in excessive damage to healthy tissue [42].

### 6.3. Summary

Despite its success in hematologic malignancies, CAR T-cell therapy needs refinement before it can be successful in solid tumors. Better selection of tumor-restricted antigens may help limit off-target effects and improve its safety profile. Ongoing work continues to optimize CAR T-cell therapy, primarily focusing on developing a more resilient and selective treatment [42,43]. Future studies should investigate CAR T-cell therapy in combination with checkpoint blockade.

## 7. Dendritic Cells

DCs are a particularly attractive immunotherapy due to their strong antigen-presenting capacity and ability to directly stimulate antigen-specific T cells that recognize and kill tumor cells. The conventional type 1 DCs (cDC1s) have shown the most promise in anti-tumor response due to their potent modulation of both innate and adaptive immune responses [47]. Moreover, the coupling of cDC1 and CD4 T cells via CD40 signaling in the presence of the proinflammatory cytokine IFN-gamma is a major source of cDC1 activation to cross-present tumor antigens to CD8 T cells in the context of MHC-I [47]. To prepare potent therapeutic autologous cDC1-based immunotherapy capable of eliciting strong anti-tumor responses, patients undergo apheresis followed by differentiation of the monocyte fraction into DCs using GM-CSF and IL-4 [48]. Subsequently, DCs are pulsed with MHC class II TAAs ex vivo and finally polarized into activated cDC1s with IFN-gamma and LPS [47,49,50].

### 7.1. Clinical Data

A recent phase I neoadjuvant clinical trial based on frontline intratumoral cDC1 immunotherapy demonstrated promising alteration in the TME and induction of tumor regression in ERBB-2+ BC patients. Intratumoral cDC1 delivery prior to milder chemotherapy demonstrated significant increases in T-cell infiltration in the tumor, specifically increased CD3+, CD4+, and CD8+ T-cells as well as B-cells, γδ T-cells, and NKT cells. Magnetic resonance imaging post-immunotherapy showed objective responses in nine patients. Seven of the twelve patients enrolled had pCR at the time of surgery (58%) [50]. Importantly, patients only experienced mild grade I-II adverse events, including fever, chills, fatigue, and injection site reactions.

### 7.2. Safety Profile and Limitations

DC therapy has shown to be safe and tolerable with mild and self-limited AEs. No patients experienced grade 3 or 4 events associated with the intratumoral therapy. However, the scalability of DC therapy outside of the clinical trial setting remains a challenge given its cost and the labor necessary to prepare patient-specific DCs.

### 7.3. Summary

Overall, DC immunotherapy is deemed to be tolerable and effective in increasing tumor immune cell infiltration that corresponds to a clinical response. Intratumoral injection of cDC1s provides a direct route to sensitizing both CD4+ and CD8+ T cells to specific antigens and does not rely on sufficient endogenous DCs. DC therapy is relatively early in its clinical use, and research to identify specific TAAs in various tumor subtypes will increase its utility in BC treatment [48,51].

## 8. The Lymph Node’s Role in Promoting Response to Immunotherapy

**Figure 1:**
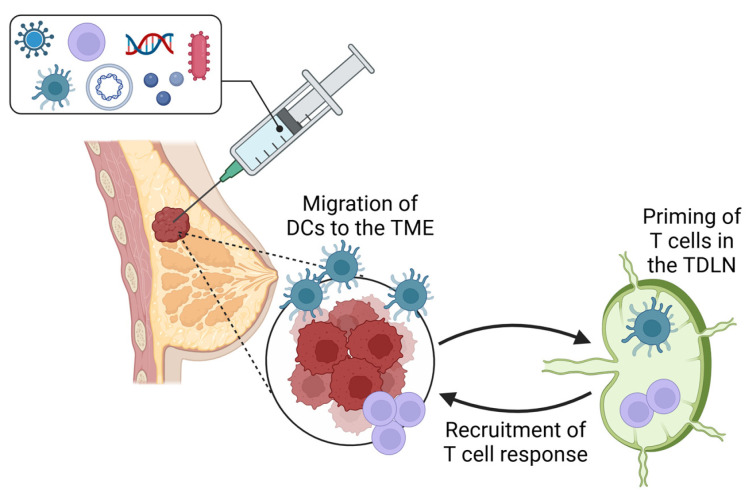
Intratumoral immunotherapies in breast cancer include oncolytic viruses, dendritic cells, CAR T cells, plasmids, DNA and RNA, innate immune agonists, and bacteria. Intratumoral injection results in migration of DCs to the TME and subsequently to the TDLN to prime the T-cell response.

A major component in the success of intratumoral immunotherapy is the activation of the TDLN to ensure adequate anti-tumor T-cell generation and subsequent tumor infiltration [52,53]. It is well-established that LNs harbor antigen-presenting cells and naïve immune cells. Migratory DCs can traffic tumor antigens to the TDLN in a CCR7-dependent manner and subsequently present the antigens to naïve lymphocytes via MHC. This will not only activate the naïve immune cells but also elicit differentiation into antigen-specific lymphocytes, thus initiating a potent tumor-specific response (Figure 1) [54].

Preclinical studies using peptide pulsed DCs have provided evidence of activation within the TDLN. TNBC and HER2+ murine models treated with peptide-pulsed DCs demonstrated increased levels of IFN-gamma secretion and antigen-specific anti-tumor CD4+ Th1 cells within the TDLN and splenocytes [48,55,56]. Evidence of a systemic immune response was demonstrated with antigen-specific CD4+ T cells within the bone marrow of treated mice [56]. Furthermore, rechallenge of treated mice did not result in tumor growth, suggesting DC priming of a specific Th1 immune response may contribute to long-term immunity [48,56]. In patients with ductal carcinoma in situ (DCIS) treated with intralesional DCs, a tumor-specific CD4+ Th1 immune response was seen in the sentinel lymph node that correlated with a higher pCR rate [49].

The role of the TDLN in response to other immunotherapies has not yet been explored in BC, although the concept has been demonstrated in melanoma. OVs induce immunogenic tumor cell death, leading to the release of TAAs and GM-CSF. These can drain to nearby TDLNs to activate DCs and subsequently prime and activate the T-cell response. Patients receiving intratumoral therapy for melanoma had enhanced immune cell infiltration, specifically CD4+ and CD8+ T cells, in the injected lesion and in non-injected lesions [57,58]. Multiple clinical trials exploring intra-dermal administration of the TLR9 agonist CpG-B demonstrated enhanced activation of DC subtypes in the sentinel lymph node and in peripheral blood. Moreover, these patients ultimately had lower numbers of positive sentinel nodes at the time of surgery and increased disease-free survival [59,60].

Altogether, preclinical and clinical data suggest that intratumoral immunotherapy has the potential to activate a robust systemic antigen-specific anti-tumor response and may play a role in preventing metastatic growth. Further work is needed to specifically address how robustly different immunotherapies activate the TDLN.

## 9. Future Perspectives

There are multiple opportunities for continued research in the development and use of intratumoral immunotherapy. Firstly, there is still a need to explore the use of immunotherapy in combination with traditional therapies and other immunotherapeutics. In other cancer types, researchers have begun exploring the combination of immunotherapy with ICIs [61,62] and chemotherapy [63]. A better understanding of how intratumoral immunotherapy remodels the TME may identify how to overcome immune escape mechanisms and thus prime the tumor for subsequent treatment like ICIs.

As discussed, the role of the TDLN in response to intratumoral immunotherapy in BC needs to be further investigated, particularly how immunotherapy can overcome the immunosuppressive environment in LNs with metastases. Emerging evidence from the use of intratumoral DCs in murine models has shown that this therapy eliminates disseminated cancer cells, thus preventing distant metastases [56]. This concept should be further explored in a clinical setting.

Finally, with the continued development and understanding of immunotherapy, its use for prevention will be at the forefront of cancer research. Preclinical models are necessary to explore the use of immunotherapeutics in halting tumor growth, particularly in high-risk populations. This will offer high-risk patients another avenue for cancer prevention and risk mitigation.

## 10. Conclusions

In conclusion, there is a wide variety of intratumoral immunotherapeutics that offer potential in the treatment of BC. Overall, intratumoral immunotherapy has not only demonstrated an acceptable safety profile but also shows evidence of local and systemic immune responses. Mostly notably, OVs and DCs appear to be the most effective in altering the TME and improving clinical responses. While nucleic acid therapy and innate immune agonists have been shown to be tolerable and somewhat immunogenic, their success ultimately relies on sufficient activation of endogenous DCs to prime T cells. Bacterial immunotherapy remains poorly understood and requires further research to prove safety and efficacy in BC. CAR T-cell therapy demonstrated an intratumoral inflammatory response; however, it lacked evidence of a corresponding clinical response. The success of OVs may be due to their dual ability to cause tumor cell lysis and activate innate and adaptive immune responses. Intratumoral cDC1s obviate the reliance on endogenous DCs and are better able to sensitize both CD4+ and CD8+ T cells to specific tumor antigens.

Overall, intratumoral immunotherapy provides a safe, feasible, and effective option for patients with BC. With continued research and increased understanding, immunotherapy offers numerous avenues to improve and excel the treatment and prevention of BC.

## Figures and Tables

**Table 1:** Ongoing or completed clinical trials.

Trial	Status	Phase	Treatment	Adjunct Treatment	Indication	Patient Enrollment	Immune Response	Clinical Response
NCT04185311	Terminated	1	T-VEC	Ipilimumab, nivolumab	TNBC or ER+/HER2− localized BC	6	↑ CD8+ T cells, cytotoxic lymphocytes, monocytes, NK cells	16.7% pCR
NCT03554044	Active, not recruiting	1b	T-VEC	Gemcitabine/carboplatin, nab-paclitaxel, paclitaxel, or endocrine therapy	HER2− (HR+/−) metastatic, unresectable, or locoregionally recurrent	20	↓ circulating lymphocytes and TIM3 expression	0% pCR, 61% partial response
NCT02779855	Active, not recruiting	1/2	Talimogene laherparepvec	Paclitaxel, doxorubicin, cyclophosphamide	Nonmetastatic TNBC	37	↑ CD3+CD8+ effector T cells, CD3+CD45RO+ memory T cells	45.9% RCB-0
NCT02658812	Terminated	2	T-VEC	None	Inoperable recurrent	11	Not available	0% response
NCT03256344	Completed	1	T-VEC	Atezolizumab	TNBC with liver metastases	36	Not available	0% complete response; 10% overall response rate
NCT02531425	Completed	1	IT-pIL-12 EP (Tavo)	Electroporation	Locally advanced or metastatic TNBC	10	↑ CD8+ TILs, ↑ expression of PD-1/PD-L1, CXCL9/10/11/CXCR3 pathways	Not available
NCT03567720	Active, not recruiting	2	Tavo	Electroporation; pembrolizumab OR pembrolizumab + nab-pacitaxel or gemcitabine plus carboplatin	Inoperable recurrent or metastatic TNBC	65	Not available	27.3% ORR
NCT03739931	Active, not recruiting	1	mRNA-2752	Durvalumab	Solid tumors including TNBC	134	↑ IL-12 and IL-36 gamma expression; ↑ IFN-gamma, TNF-alpha, PD-L1, T-cell infiltration	Not available in TNBC
NCT03052205	Completed	1b	IMO-2125 (TLR9 agonist)	None	Refractory solid tumors	54	↑ MHC I and II expression, IFN-gamma expression	Not available
NCT04144140	Terminated	1	E7766 (STING agonist)	None	Advanced solid tumors	24	Not available	Not available
NCT01924689	Completed	1	Clostridium *noyvi*-NT	None	Advanced solid tumors	24	Overall, 3 patients with increased T-cell infiltration	Of BC pts, 1 pt with stable disease, 1 unevaluable
NCT03435952	Active, not recruiting	1	Clostridium *noyvi*-NT	Pembrolizumab, doxycycline	Advanced solid tumors	16	Not available	25% ORR
NCT01837602	Completed	1	c-Met-CAR T-cells	None	Metastatic	6	↑ CD4+ T cell and mononuclear immune cells	Not available
NCT05325632	Completed	1	cDC1	Trastuzumab, pertuzumab	ERBB2+ breast cancer	12	↑ CD3+, CD4+, CD8+ T cells, B-cells, NKT cells	58% pCR

↑: increased; ↓: decreased

## Abbreviations

The following abbreviations are used in this manuscript:

AE	adverse event
APC	antigen-presenting cell
BC	breast cancer
CAR	chimeric antigen receptor
cDC1	conventional type 1 dendritic cell
DC	dendritic cell
DCIS	ductal carcinoma in situ
DNA	deoxyribonucleic acid
EGFR	epidermal growth factor receptor
GM-CSF	granulocyte–macrophage colony-stimulating factor
HER	human epidermal growth factor
HSV	herpes simplex virus
ICI	immune checkpoint inhibitor
IFN	interferon
MDSC	myeloid-derived suppressor cells
MHC	major histocompatibility complex
mRNA	messenger ribonucleic acid
NK	natural killer
NKT	natural killer T cell
OV	oncolytic virus
PAMP	pathogen-associated molecular pattern
pCR	pathologic complete response
PD	programmed death protein
PDL	programmed death ligand
PMN	polymorphonuclear leukocyte
RCB	residual cancer burden
RNA	ribonucleic acid
STING	stimulator of interferon gamma
TAA	tumor-associated antigen
TAM	tumor-associated macrophage
TDLN	tumor-draining lymph node
TIL	tumor-infiltrating lymphocytes
TLR	toll-like receptor
TLS	tertiary lymphoid structure
TME	tumor microenvironment
TNBC	triple-negative breast cancer
TVEC	talimogene laherparepvec
